# Reattachment of a Dehydrated Tooth Fragment Using Retentive Holes

**DOI:** 10.7759/cureus.6640

**Published:** 2020-01-13

**Authors:** Faisal A AlQhtani

**Affiliations:** 1 Pediatric Dentistry, Ministry of Health, Riyadh, SAU

**Keywords:** dental trauma, reattachment, resin cement, tooth fragment, retentive hole

## Abstract

Coronal fracture of the anterior teeth is mainly common among children and adolescents. Though diverse treatment modalities are available, tooth fragment reattachment is generally considered a viable treatment option due to simplicity, natural aesthetics, and functional success. This paper presents a case of a 10.5-year-old female patient with a fractured maxillary central incisor with a dehydrated fragment. The dehydrated fragmented part was reattached with the help of retentive holes using an adhesive bonding agent and a resin composite cement. Follow-up at 15 months showed that the tooth was vital and functional with natural aesthetics.

## Introduction

Dental trauma can be caused due to accidental falls or sports injuries such as football, baseball, basketball, and hockey [1]. Chewing and munching on hard foods and objects such as nuts, frozen ice cubes, and candies can also lead to tooth crack or tooth fracture [2]. Dental trauma might have a severe influence on the social and psychological life of a patient [3]. Of all traumas to the dental hard tissues, coronal fractures of permanent incisors represent 18-22%. Out of these 18-22% fractures, 28-44% are simple (enamel + dentin) and 11-15% are complex (enamel +dentin +pulp). Of these fractures, 96% involve maxillary central incisors [4].

Dental trauma is one of the common injuries affecting people of all ages, particularly children under the age of 18 years. Since most of the damage to the teeth happens in younger people [4], the treatment of these damages should be done in the right perspective by ensuring long-term benefits.

With advanced dental technology, many types of restorative methods and materials are available. The treatment option to restore traumatized teeth may include direct and indirect restorative procedures. Choosing direct or indirect restoration depends on the nature of the tooth issue and extent. For a restorative dentist, the assessment and selection process has its own challenge.

Tooth fragment bonding has become popular due to its various advantages such as anatomical features, color, and surface appearance. It can furnish positive psychological response and long-lasting aesthetics [5]. Bonding is one of the simplest and least expensive procedures, and some dentists describe it as the best method to make corrections of teeth in low bite pressure areas. This article describes the bonding of tooth fragment in a 10.5-year-old child with no serious breakage or pulp exposure.

## Case presentation

**Figure 1:**
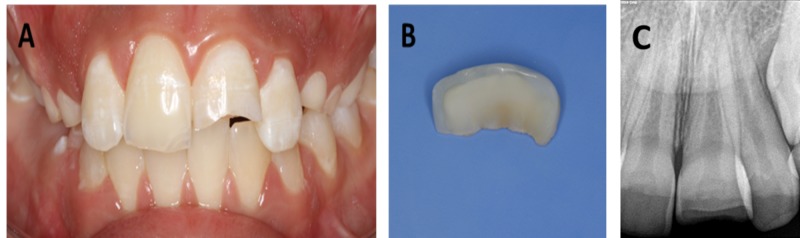
Pre-operative images. (A) Fractured maxillary central incisor. (B) Fractured fragment of the maxillary central incisor. (C) Peri-apical radiograph of the broken maxillary central incisor.

A 10.5-year-old child presented along with her father after falling down the stairs two days before and having one of her front central tooth broken (#21), as shown in Figure 1A. She brought the fragment of the broken tooth (Figure 1B) stored in a plastic container in order to get the fragment glued back on the tooth and was complaining tooth sensitivity while exposing to air and drinking.

A comprehensive intraoral and radiographical examination (Figure 1C) was performed to diagnose, locate, and measure the extent of tooth breakage. Clinical examination revealed a class II fracture. It was found that tooth #21 was intact and immobile after fracturing, with no sign of gingival inflammation. A vitality test was conducted to evaluate the blood supply to the tooth, and a sensitivity test (thermal cold test) was performed to assess the sensory response. The outcomes were positive, and a normal response was noticed.

Fragment of tooth #21 was cleaned and checked with the broken tooth in order to ensure that no part was lost. The fragment was in good condition and fit reasonably well on the fractured tooth. However, the perceptible shade difference was observed between the broken tooth and the fragment due to the dehydration of the broken fragment during the last two days. Both the father and daughter were informed about the difference in hue and shade. On their consent to proceed with the reattachment procedure, the fragment was stored in saline (for one hour) until reattached with the tooth.

**Figure 2:**
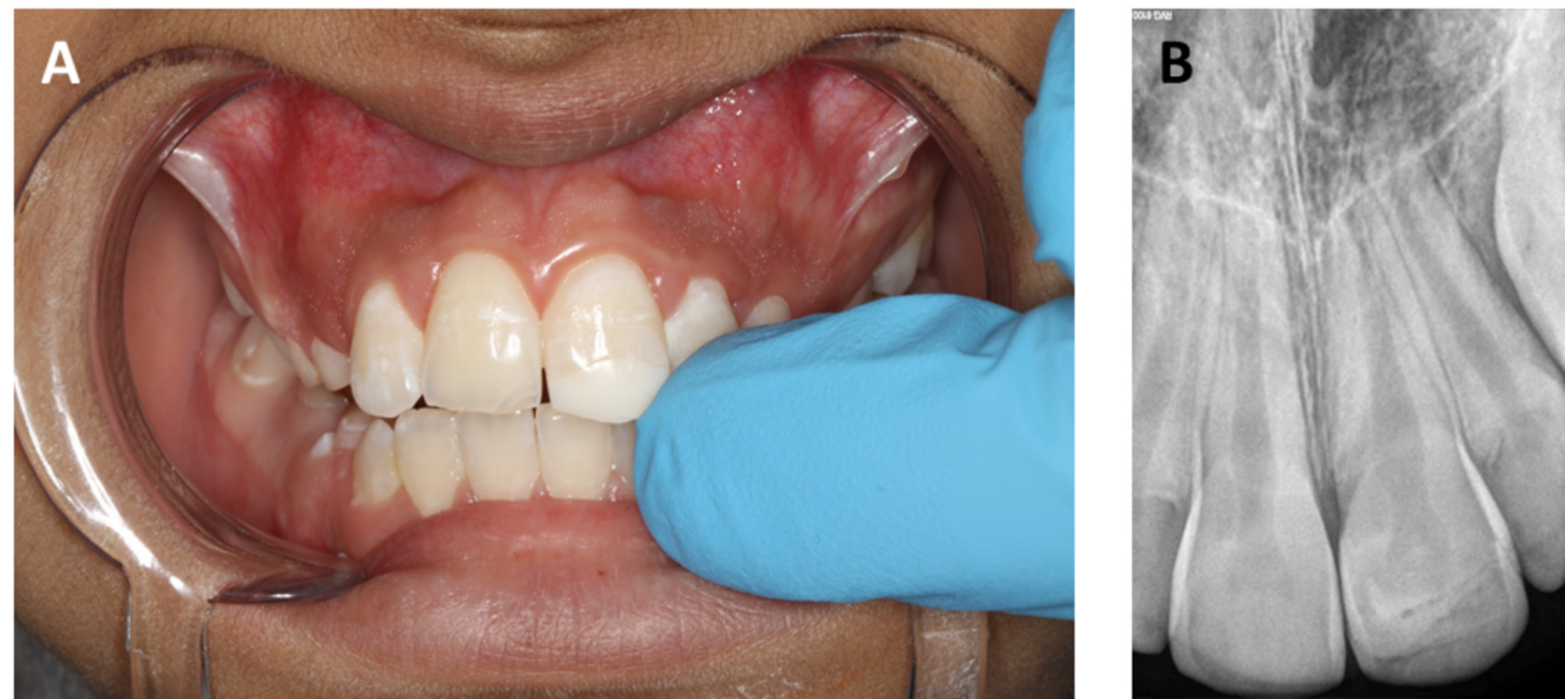
Post-operative image and radiograph. (A) Reattachment of the broken tooth fragment in the maxillary central incisor. (B) Peri-apical radiograph immediately after the attachment of the broken fragment.

Topical and local anesthesia was administered to the patient. Bevels were created on a broken tooth to help in increased retention. The beveling was performed from the palatal as well as the buccal surfaces. Vitrebond (3M-ESPE, St. Paul, MN, USA) was used to fix the retentive holes into the dentin. Next, a self-etching primer (Clearﬁl Liner Bond SE Primer, Kuraray, Osaka, Japan) was applied for 20 seconds and light-cured according to the manufacturer’s instructions. Next, a broken tooth and a fragment were air-dried gently. Dual cure flowable composite (SmartCem2, Dentsply Maillefer, USA) with A2 shade was applied on the broken incisal edge of the tooth and the fragment. The fragment was positioned accurately and photopolymerized for 20 seconds each from the labial and palatal sides. After successful completion of the procedure, instructions were given to the father and patient to avoid hard foods (Figure 2).

Six-week follow-up after treatment

The patient was recalled for a follow-up after six weeks. A peri-apical radiograph was taken to examine the lamina dura, periodontal ligament space, and bone socket. Vitality of pulp and blood flow was determined by a pulp sensibility test. The findings were positive, and the tooth was functioning normally.

Nine-month follow-up after treatment

Clinical examination was performed at the nine-month follow-up. Vitality tests were again repeated. The findings were positive, and the tooth was functioning normally with no pulpal or peri-apical changes.

Fifteen-month follow-up after treatment

**Figure 3:**
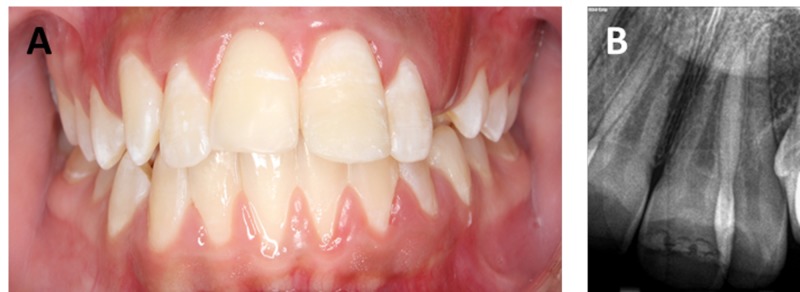
Post-operative follow-up images at 15 months. (A) Functional maxillary central incisor. (B) Peri-apical radiograph showing no sign of pulpal or per-apical changes.

Again, follow‑up examinations were carried out at a 15‑month interval, and the tooth was functioning normally and esthetic was pleasing (Figure 3). The difference in shade had become unnoticeable.

## Discussion

Modern dentistry has become more refined. It has enabled us to do things that were never thought to be possible. In modern dentistry, cosmetic dentistry is gaining widespread popularity as it has assisted in improving the appearance of the teeth, mouth, and smile of a common man.

The tooth fragment bonding technique is considered to be conservative; however, with the advancement in adhesive dentistry, the practice of fragment reattachment has become easier and reliable [6]. Though tooth fragment bonding provides a number of distinct advantages, the main advantage is aesthetics, which brings satisfaction [7].

Rehydration and dehydration periods can produce a notable effect on the bonding strength of the fragment [8]. Some analysts believe that keeping the fragment in water for 24 hours prior to reattachment increases the bond strength [9]. Although dried tooth fragment was successfully glued to a broken tooth, the tooth was functional even at 15-month follow-up. However, public awareness is important regarding emergency management of dental trauma as patients present to hospitals and clinics with cracking/breaking of their teeth. Milk and saliva environment are some of the recommended storage media that has been suggested for keeping fragment during the extra-oral time [10]. Study shows that by keeping fragment either in milk or saliva environment for 24 hours prior to reattachment can significantly enhance bond strength as compared with water [10]. The reason behind this is that milk contains calcium and phosphate, which make dentin surface harden and stiffen, enabling a better bond strength. However, in this case, a fragment was successfully glued without storing the fragment in any medium prior to bonding. The fragment was hardly stored in saline for one hour before reattachment with the tooth using retentive holes. Interestingly, the broken fragment was mismatching in hue and shade of the broken tooth at the time of bonding; however, at 15-month follow-up, there was no sign of mismatching. This might suggest that during the clinical life, the broken fragment absorbed water and stains thus the hue and the shade of fragment eventually matched with the tooth.

There are many techniques for reattaching the fragment; however, studies show that the original mechanical strength of the tooth cannot be restored to its full [11]. Nevertheless, it is possible to retain a reattached tooth for a long period of time with some mechanical resistance. Once a broken tooth fragment is bonded back in place, prevention measures should be adopted to avoid tooth trauma.

## Conclusions

Reattachment is a viable treatment option for restoring aesthetics and functional success. The clinical examination at a 15-month follow-up demonstrated that using retentive holes on class II fracture could be a viable option for the reattachment of the dehydrated broken fragment provided that hard food is avoided. Moreover, the 15-month clinical life of the dehydrated tooth fragment caused the shade difference completely unnoticeable.
