# Correction: Reattachment of a Dehydrated Tooth Fragment Using Retentive Holes

**DOI:** 10.7759/cureus.c28

**Published:** 2020-01-16

**Authors:** Faisal A AlQhtani

**Affiliations:** 1 Pediatric Dentistry, Ministry of Health, Riyadh, SAU

The author mistakenly used the word "screws" instead of "holes" throughout the article, including the title. These incorrect instances of "screws" have been replaced with the correct word ("holes"). In addition, the following sentence was removed as it was also mistakenly included by the author: "Furthermore, retentive holes were made and screws were placed into the dentin to retain the fragment in position."

The Cureus editorial team deeply regrets that these errors were not caught prior to publication.

